# Efficient Degradation of Alginate and Preparation of Alginate Oligosaccharides by a Novel Biofunctional Alginate Lyase with High Activity and Excellent Thermophilic Features

**DOI:** 10.3390/md21030180

**Published:** 2023-03-14

**Authors:** Li Li, Shengsheng Cao, Benwei Zhu, Zhong Yao, Bo Zhu, Yimin Qin, Jinju Jiang

**Affiliations:** 1 College of Food Science and Light Industry, Nanjing Tech University, Nanjing 211816, China; 2 Key Laboratory of Seaweed Fertilizers, Ministry of Agriculture and Rural Affairs, Qingdao Brightmoon Seaweed Group Co., Ltd., Qingdao 266400, China

**Keywords:** alginate, alginate lyase, high catalytic activity, thermal stability, biofuels

## Abstract

The enzymatic degradation of seaweed polysaccharides is gaining interest for its potential in the production of functional oligosaccharides and fermentable sugars. Herein, a novel alginate lyase, AlyRm3, was cloned from a marine strain, *Rhodothermus marinus* DSM 4252. The AlyRm3 showed optimal activity (37,315.08 U/mg) at 70 °C and pH 8.0, with the sodium alginate used as a substrate. Noticeably, AlyRm3 was stable at 65 °C and also exhibited 30% of maximal activity at 90 °C. These results indicated that AlyRm3 is a thermophilic alginate lyase that efficiently degrades alginate at high industrial temperatures (>60 °C). The FPLC and ESI−MS analyses suggested that AlyRm3 primarily released disaccharides and trisaccharides from the alginate, polyM, and polyG in an endolytic manner. In the saccharification process of sodium alginate (0.5%, *w*/*v*), the AlyRm3 yielded numerous reducing sugars (1.73 g/L) after 2 h of reaction. These results indicated that AlyRm3 has a high enzymatic capacity for saccharifying the alginate, and could be used to saccharify the alginate biomass before the main fermentation process for biofuels. These properties make AlyRm3 a valuable candidate for both fundamental research and industrial applications.

## 1. Introduction

Alginate is a linear polysaccharide composed by (1→4)-linked β-D-mannuronic acid (M) and α-L-guluronic acid (G) residues [1]. It linked these units in three kinds of different blocks, namely poly β-D-mannuronic acid (polyM), poly α-L-guluronic acid (polyG), and the heteropolymer (polyMG). Alginate is widely found in brown algae, and its content is approximately 30–60% dry weight. In addition, alginate also exists in some red algae and can be produced by several bacteria, such as the genera *Azotobacter* and *Pseudomonas* [2]. The abundance of these species has made alginate an attractive and important source of renewable biomass for biofuel production [3]. Today, alginate is mainly utilized for biomedical (e.g., 3D-hydrogels and 2D membranes for soft tissue regeneration), environmental (e.g., sponges for water purification), and food (e.g., rheological additives) applications [4,5,6,7,8]. Thus, the enzymatic degradation of alginate is of significant biotechnological importance.

Alginate lyases discovered to date have been isolated, cloned, and characterized from marine and/or soil bacteria and fungi, Chlorella viruses, marine algae, and marine invertebrates [9]. In the Carbohydrate-Active enZYmes database (http://www.cazy.org/, accessed date: 10 September 2022), alginate lyases belong to fourteen PL families: PL5, 6, 7, 8, 14, 15, 17, 18, 31, 32, 34, 36, 39, and 41 [10], implying substantial structural diversity of catalytic sites [11]. Most alginate lyases show different substrate specificity for four different glycosidic bonds (M-M, G-G, M-G, and G-M) in alginate molecules. As per this feature, alginate lyases can be categorized into three classes: polyM-specific (EC 4.2.2.3), polyG-specific (EC 4.2.2.11), and bifunctional lyases (EC 4.2.2.-) [10]. Alternatively, alginate lyases can also be divided into endo- and exo-type lyases based on their action patterns. Endo-type lyases degrade the inside-chain glycosidic bonds to create alginate oligosaccharides (AOS) with various DPs, while exo-type lyases can only produce monomers or dimers by gradual degradation from the end of alginate polymers [12].

Concerns about bioethanol production due to its impact on the food chain, in recent years, have driven the search for more sustainable production of bioethanol based on non-food biomass, such as lignocellulosic or algal biomass. Brown algae are considered a renewable biomass for biofuel ethanol production due to their high growth rate and high sugar content [13]. The collection of brown algal biomass is relatively easy due to the lack of lignin. Bioethanol from algal biomass is receiving growing interest in relation to the search for more sustainable processes of renewable energy production [14]. The realization of converting alginate into fuels requires two processes: (1) alginates are depolymerized efficiently into AOS; (2) metabolically engineered microorganisms assimilate alginate degradation products into biofuels [15]. Alginate lyases from marine extremophiles are among the most promising biocatalysts due to their resistance to temperature, salt concentration, and contaminants. In the process of alginate lyase catalysis, the catalytic reaction can be carried out at higher temperatures due to the existence of thermal stability, and the viscosity of the reaction mixture is reduced and the enzymatic activity is improved, thereby promoting the conversion of the substrate [14]. Therefore, alginate lyase with good thermal stability is of great value for the enzymatic production of alginate oligosaccharides. Magnus Ø. Arntzen et al. have described a novel, thermostable exolytic alginate lyase (AMOR_PL17A), whose gene was retrieved from a metagenomic data set collected from the Arctic Mid-Ocean Ridge (AMOR). They showed that the enzyme has a broad pH optimum in the region of pH 5.0 to 6.0, and it was stable for 24 h at 60 °C. This enzyme is promising for the production of biofuels since it afforded high yields of alginate degradation [16].

In this study, we have cloned and expressed a novel, thermostable exolytic alginate lyase-AlyRm3 from *Rhodothermus marinus* DSM 4252, which can degrade alginate into alginate oligosaccharides with high efficiency. The biochemical characterization and action pattern of AlyRm3 were investigated. Compared to other alginate lyases, AlyRm3 exhibited excellent high thermal stability and high capability for saccharifying the sodium alginate, releasing high amounts of di-, tri-, and tetra-saccharides from sodium alginate. This study is expected to provide efficient enzymes for the degradation of alginate and the production of biofuels from algal crops.

## 2. Results

### 2.1. Sequence Analysis

The open reading frame (ORF) of AlyRm3 (GenBank accession. CP001807) consists of a 2613 bp gene encoding 870 amino acids with a theoretical molecular weight of 96.62 kDa and a 16-amino acid signal peptide at the N-terminus. As shown in Figure S1a, the full-length enzyme contains two domains: a Hepar_II_III domain (His397-Thr585, pfam07940) and an FlgD-ig domain (Ser793-Ala861, pfam13860). The PL39 alginate lyase Dp0100 (GenBank accession no. MK628724.1) from *Defluviitalea phaphyphila* suggests that the catalytic site of AlyRm3 is a block of sequence similar to the Hepar_II_III region of the heparinase II/III enzymes [17]. Transmembrane helices have been predicted in AlyRm3 to adopt the topology shown in Figure S1b. The phylogenetic tree was constructed and it exhibited AlyRm3 clusters with several alginate lyases of the PL39 family (Figure S1c).

As the sequence alignments are shown in Figure S2, AlyRm3 has the highest sequence identity (40%) with Dp0100 [17]. Additionally, AlyRm3 contained the conserved regions such as “ADNH”, “PRPHNH”, “RYLLF”, and “YREG”, which were involved in substrate binding and catalytic activity. Two key residues Y244 and H405 in the catalytic mechanism predicted to be involved in the catalytic process based on sequence alignment were also conserved in PL39 alginate lyases.

### 2.2. Expression and Purification of AlyRm3

**Figure 1:**
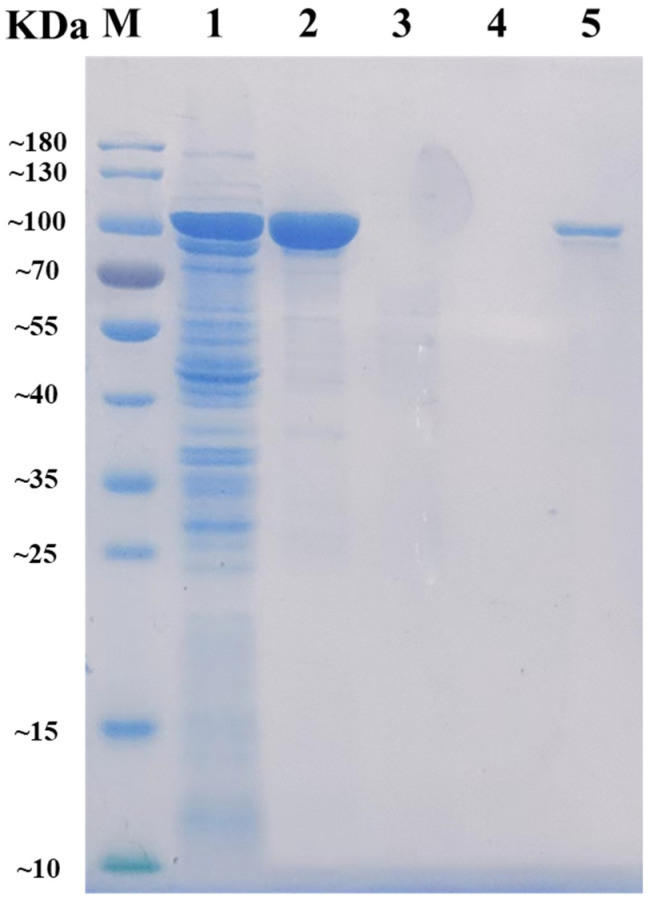
SDS-PAGE analysis of the molecular mass and purification effect of AlyRm3. Lane M protein: restrained marker (Vazyme, Nanjing, China); lane 1: induced cell lysate of E. coli-pET21a-AlyRm3; lane 2: renatured purified AlyRm3; lane 3: binding buffer during purification; lane 4: wash buffer during purification; lane 5: elution buffer during purification.

After heterologously expressed and purified by Ni-NTA Sepharose affinity chromatography, the recombinant AlyRm3 was obtained and then analyzed by SDS-PAGE. As shown in Figure 1, the band of the target protein AlyRm3 is located between 70 kDa and 100 kDa, which is consistent with the predicted molecular mass of 96.62 kDa. The alginate lyase activity of AlyRm3 was verified with an enzymatic activity assay, demonstrating that AlyRm3 is an alginate lyase.

Currently, in the CAZY database, Dp0100, belonging to the PL39 family, is the only enzyme that does not contain an alginate lyase domain but still has alginate lyase activity. Ji et al. constructed a series of Dp0100 cutting mutants and demonstrated that the combination of the DUF4962 domain and Hepar_II_III region were necessary for the alginate lyase activity of Dp0100 [17]. Thus, it can speculate reasonably that the Hepar_II_III domain plays a key role in AlyRm3 to require for alginate lyase activity. AlyRm3 is a novel alginate lyase with no alginate lyase domain.

Three different substrates (0.5% sodium alginate, 0.5% polyM, and 0.5% polyG) were applied to determine the activities of AlyRm3. As shown in Table 1, the AlyRm3 exhibited higher activities towards sodium alginate (37,315.08 U/mg) than towards polyM (28,814.31 U/mg), and polyG (21,329.21 U/mg). Thus, AlyRm3 is a new PL39 alginate lyase with a preference for sodium alginate, and can degrade polyG and polyM with the same activity. Furthermore, the *K_m_* values of AlyRm3 with sodium alginate, polyM, and polyG as substrates were 15.6738, 7.3264, and 5.3961 mM, respectively. Additionally, the *K_cat_* values of AlyRm3 toward alginate, polyM, and polyG were 141.69, 2323.78, and 212.07 s^−1^, respectively. AlyRm3 is considered to have the strongest affinity for the G-G blocks. It suggested that AlyRm3 exhibits a higher catalytic efficiency towards M-blocks than that towards G-blocks and MG-blocks. Although the activities of AlyRm3 on polyG and polyM are only half of that on sodium alginate, the catalytic efficiency is higher. The characteristics of some alginate lyases with high activity are summarized (Table S1).

### 2.3. Biochemical Characterization of AlyRm3

**Figure 2:**
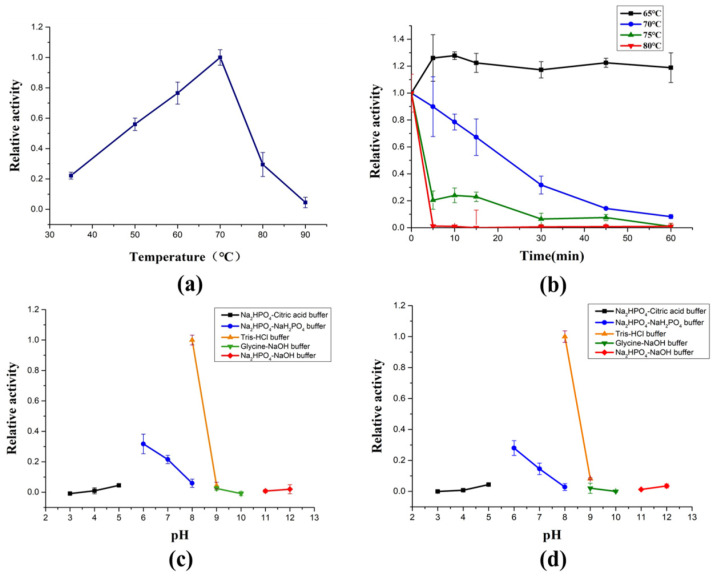
Biochemical characterization of AlyRm3. (**a**) The optimal temperature of AlyRm3. (**b**) The temperature stability of AlyRm3. All reactions in (**a**,**b**) were carried out at pH 8.0 (Tris-HCl buffer). (**c**) The optimal pH of AlyRm3. (**d**) The pH stability of AlyRm3.

AlyRm3 exhibits surprising thermophilic features, showing maximum activity at 70 °C (Figure 2a) and retaining almost all of its maximum activity after 1 h incubation at 60 °C (Figure 2b). Moreover, enzymes with high-temperature stability and high activity are rarely reported. To highlight the potential superiority of AlyRm3 application under high-temperature industrial conditions, several thermophilic enzymes were compared (Table S2). So far, there are few studies on the thermostability mechanism of alginate lyase. According to previous reports, the thermostability of rNitAly is related to the disulfide bond formed between Cys80 and Cys232 [18]. The thermal stability of AlgC-PL7 from *Cobetia* sp. NAP1 may be related to the α-helix [19]. The thermal stability of AlyM may be related to the compactness of the enzyme [20]. The thermostability of AMOR-PL17A may be related to the more prolines, arginines, phenylalanines, and glutamines in the protein sequence and have a more overall positive charge [16]. Based on the analysis of the protein sequence and secondary structure of AlyRm3, it is speculated that the reason for the thermostability of AlyRm3 may be that its α-helix supports the rigid structure of the protein.

The optimal pH of AlyRm3 is 8.0 (Tris-HCl buffer) (Figure 2c), but its activity decreased sharply in the acidic and strongly alkaline range. AlyRm3 retained almost all of its maximal activities after being incubated at the same pH for 24 h (Figure 2d).

### 2.4. Effect of Metal Ions on AlyRm3

**Figure 3:**
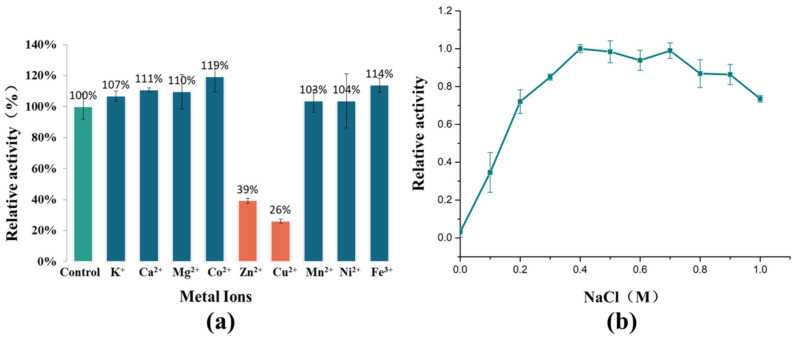
The effects of metal ions and NaCl on the activity of AlyRm3. (**a**) The effects of metal ions on the activity of AlyRm3. (**b**) The effects of NaCl on the activity of AlyRm3.

A study on the effect of metal ions on the enzymatic activity of AlyRm3 has also been completed (Figure 3a). K^+^, Ca^2+^, Mg^2+^, Co^2+^, and Fe^3+^ can significantly activate the activity of AlyRm3, while Zn^2+^ and Cu^2+^ inhibit the activity of AlyRm3. In summary, AlyRm3 showed good tolerance to metal ions compared with other alginate lyases.

In order to adapt to the high sodium environment of the ocean, many alginate lyases possess salt tolerance and/or salt activation properties. For example, rTsAly7A derived from *Thalassomonas* sp. LD5 has shown the highest activity at 100 mM NaCl and was twice as active as it was at 0m NaCl [21]. One study has shown that the salt activation mechanism of AlyC3 is to preserve a dimeric quaternary structure. Salt activation of AlyPM from *Pseudoalteromonas* sp. SM0524 is due to the enhanced affinity of the substrate for NaCl [22]. The reason for the salt-activating properties of AlgM4 may be that NaCl alters its secondary structure, eventually leading to substrate affinity and resistance to thermal denaturation [23]. As shown in Figure 3b, recombinant AlyRm3 showed weak enzymatic activity at 0 M NaCl. As the NaCl concentration increased, the enzymatic activity showed first a sharp increase (0–0.2 M), and showed the highest activity at 0.4 M NaCl. Finally, when the concentration of NaCl > 0.7 M, the enzymatic activity started to decrease slowly. The above experimental data demonstrated that AlyRm3 is a novel salt-activated alginate lyase. The optimum NaCl concentration is 0.4 M. This result is consistent with the optimum growth environment of *Rhodothermus marinus* DSM 4252. More studies are required to explore the salt activation mechanism of AlyRm3.

### 2.5. Products Distribution and Action Pattern of AlyRm3

**Figure 4:**
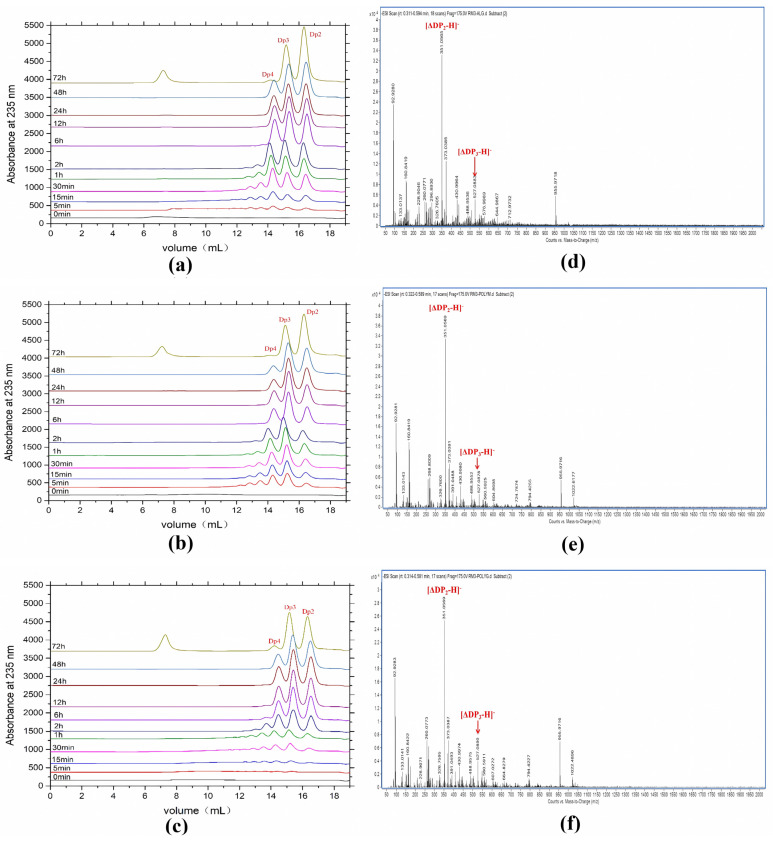
FPLC analysis (**a**−**c**) and ESI−MS analysis (**d**,**e**) of the degradation products for 72 h. (**a**) sodium alginate was used as the substrate. (**b**) polyM was used as the substrate. (**c**) polyG was used as the substrate. (**d**) sodium alginate was used as the substrate. (**e**) polyM was used as the substrate. (**f**) polyG was used as the substrate.

Degradation products of AlyRm3 at various times (0–72 h) were analyzed by Fast protein liquid chromatography (FPLC). As shown in Figure 4a–c, AlyRm3 could degrade the three substrates into tetrasaccharide, trisaccharide, and disaccharide during the initial stage of the reaction. However, tetrasaccharide was degraded into trisaccharide and disaccharide after incubation for 72 h.

**Figure 5:**
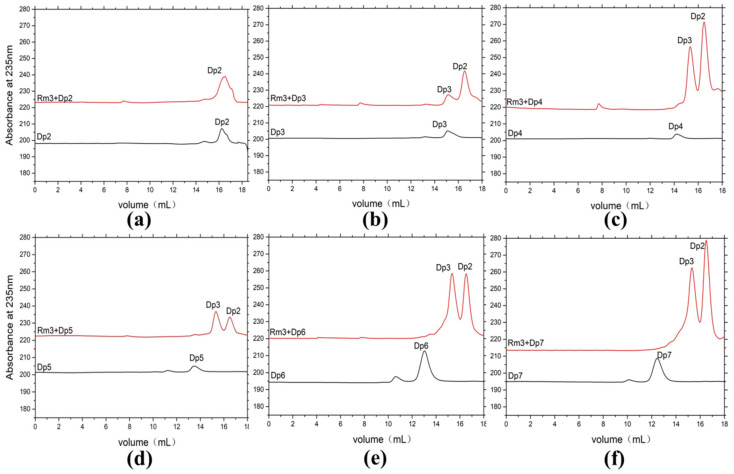
FPLC analysis of the degradation products of AlyRm3 on sodium D-mannuronic acid with different degrees of polymerization (**a**–**f**) (DP2-7). The black line shows the substrate with different DPs, and the red line shows the mixture after the reaction of the substrate with AlyRm3.

ESI-MS was used to analyze the composition of the end products (Figure 4d,e). When sodium alginate, polyM, and polyG are used as substrates, oligomers of trisaccharide and disaccharide (Signals of 351.05 *m/z* [ΔDP2-H]^−^, and 527.08 *m/z* [ΔDP3-H]^−^) are released as end products. We further confirmed the action mode of AlyRm3 by studying the degradation products (48 h) of AlyRm3 on sodium D-mannuronic acid with different degrees of polymerization (DP2-7) using FPLC. The results showed that AlyRm3 could degrade oligosaccharides (DP > 2) into trisaccharides and disaccharides (Figure 5). As can be seen from the above results, AlyRm3 can efficiently degrade three substrates into trisaccharide and disaccharide with endo mode.

### 2.6. Molecular Modeling

**Figure 6:**
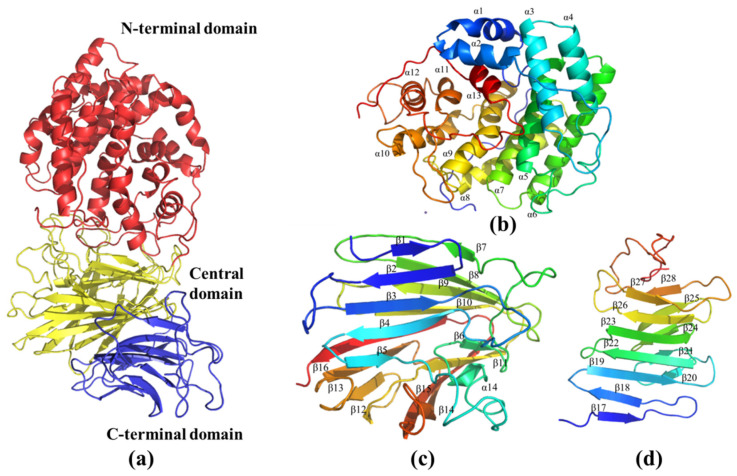
The three-dimensional model of AlyRm3. (**a**) Schematic representation of the structure of AlyRm3 showing its N-terminal (red), central (yellow), and C-terminal domains (blue). (**b**) Schematic diagrams of the N-terminal domain (blue to red from the N to C termini). (**c**) Schematic diagrams of the central domains. (**d**) Schematic diagrams of the C-terminal domains.

The three-dimensional model of AlyRm3 was constructed with PHYRE2 based on the homologous structure of Dp0100 (PDB: 6JPN) of *Defluviitalea phaphyphila*. The sequence identity between AlyRm3 and Dp0100 was high (40%). Correspondingly, the protein model was successfully constructed with a confidence level of 100%. As shown in Figure 6a, the overall structure of AlyRm3 can be divided into three domains. The N-terminal structural domain is predominantly helical in structure, formed by an incomplete (α/α)_6_ toroid (Figure 6b). The central structural domain consists of 16 antiparallel β-strands arranged in two β-sheets with a twisted α-helix. (Figure 6c). The C-terminal structural domain is a typical β-sandwich that consists of two anti-parallel β-sheets including 12 β-strands (Figure 6d). The two faces of the central structural domain are immediately adjacent to the N-terminal structural domain and C-terminal structural domain, respectively, and form a four-layered β-sheet stack with the C-terminal structural domain (Figure 6a).

**Figure 7:**
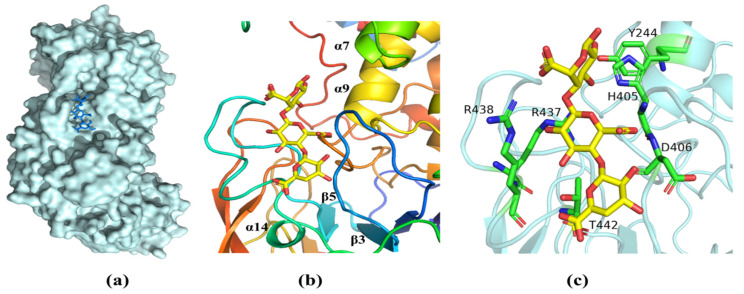
The substrate-binding groove of AlyRm3. (**a**) Surface representation of the binding site of ΔMG in AlyRm3 (indicated by the stick, drawn in blue). (**b**) Schematic representation of the binding site in ΔMG (shown as sticks, drawn in yellow) and surrounding secondary structures (colored as in Figure 6). (**c**) Key residues (drawn in green) interacting with the ΔMG (shown as sticks and drawn in yellow).

Analysis of the structure of the complex with ΔMG shows that the oligosaccharide was bound in a long cleft formed between the N-terminal domain and the central domain (Figure 7a). One side of the substrate binding groove is made up of the loop between β3 and β4 (Figure 7b). The other side is the loop between β5 and α14. We note that the open ends of the substrate-binding cleft give it a grooved appearance, so that the enzyme can accommodate longer chains of oligosaccharide molecules more easily. (Figure 7a). Crucial interactions between the AlyRm3 and the oligosaccharide involve the recognition of the C5 carboxyl moiety on each of the uronic acid residues, which alternately direct to the opposite side of the substrate-binding site (R438, R437, T442, D406, H405, and Y244) (Figure 7c). In particular, H405 and Y244 act as the catalytic base and catalytic acid, respectively.

### 2.7. Saccharification of Alginate by AlyRm3

**Figure 8:**
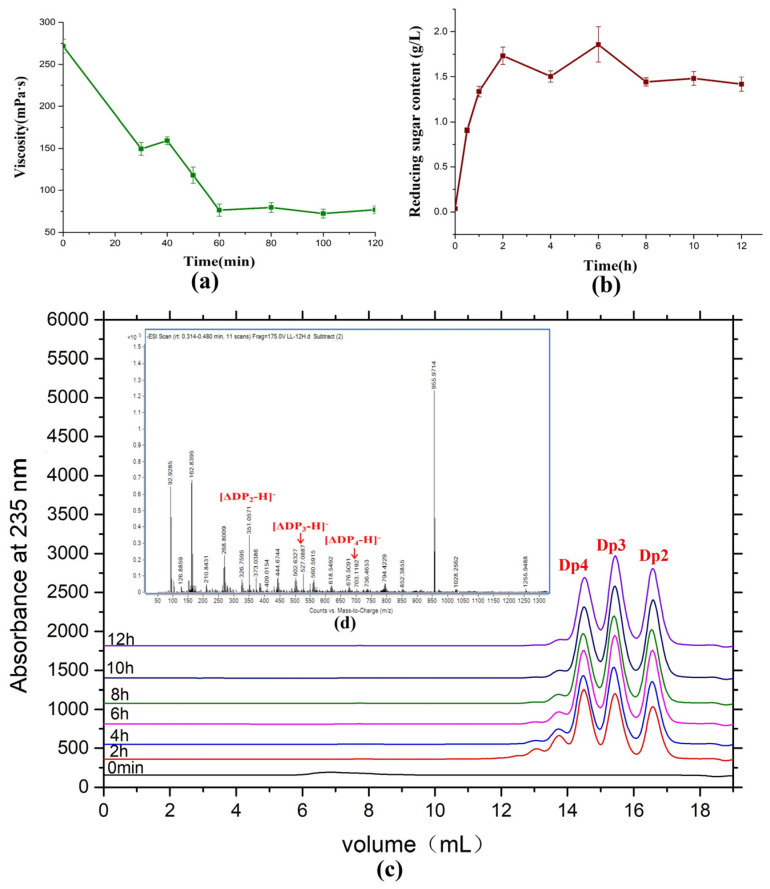
Saccharification of alginate with AlyRm3. (**a**) Change in viscosity at various time points during the AlyRm3 degradation reaction. (**b**) Change in reduced sugar at various time points during the AlyRm3 degradation reaction. (**c**) FPLC analysis was performed on the degradation products of saccharified alginate for 12 h. (**d**) ESI−MS analysis was performed on the degradation products of saccharified alginate for 12 h.

Saccharification sodium alginate using AlyRm3 was used to evaluate the potential of industrial preparation of alginate oligosaccharides by AlyRm3 (Figure 8). During the reaction, the viscosity of sodium alginate decreased sharply within the first 60 min and then maintained at about 75 mPa∙s (Figure 8a). The concentration of reducing sugar reached its maximum when the reaction had been going on for two hours and then remained constant (Figure 8b). In addition, by analyzing the products of the saccharification experiments, the products after 6 h were mainly disaccharides, trisaccharides, and tetrasaccharides, and the content of the three oligosaccharides was basically the same (Figure 8c,d). It demonstrated that AlyRm3 is a powerful tool for the industrial production of alginate oligosaccharides.

## 3. Discussion

Extreme enzymes, which are enzymes produced by microorganisms living in extreme environments, have attracted a lot of attention in recent years, due to their wide range of applications in various fields such as the food and pharmaceutical industries, bioenergy (ethanol, hydrogen), and bioremediation of contaminated areas. As marine extreme enzymes have adapted themselves to harsh environmental conditions, they can retain activity in many typical industrial environments, such as extreme temperatures and pH, high salt concentrations, and in the presence of metal ions and organic solvents. It is foreseeable that marine extreme enzymes will become important biological tools for various industrial fields in the future.

Due to the great diversity of marine microorganisms, extreme alginate lyases have not been fully explored to date. Therefore, the identification of novel enzyme-producing species and novel extreme alginate lyases is a key issue for the utilization of alginate lyases in modern biotechnology. In this study, we report on a novel thermophilic alginate lyase and explain its degradation mode for efficient production of alginate oligosaccharides. AlyRm3 is the second enzyme in the PL39 family to be reported and has the highest homology of 40% with Dp0100. AlyRm3 has a typical PL39 family (α/α)_n_ barrel + antiparallel β-fold structure. We predicted that the conserved motifs “ADNH”, “PRPHNH”, “RYLLF”, and “YREG” may play a catalytic and substrate binding role in the PL39 family alginate lyases. The AlyRm3 showed optimal activity at 70 °C and pH 8.0. Noticeably, AlyRm3 was stable at 65 °C and also showed some activity at 90 °C. Compared with other thermostable alginate lyases that have been characterized so far (Table S2), the optimum temperature of AlyRm3 is comparable to that of rNitAly. However, AlyRm3 is second only to AMOR_PL17A, and far superior to other lyases in terms of temperature stability. More remarkably, AlyRM3 showed an enzymatic activity of up to 37,315.08 U/mg against sodium alginate. Among the characterized alginate lyases that have been introduced as having a high activity (Table S1), AlyRm3 has an enzymatic activity several times or even tens of times higher than other alginate lyases for the same definition of activity. AlyRm3 shows little activity in the absence of Na^+^. Therefore, AlyRm3 is a typical Na^+^ activating enzyme. AlyRm3 has a good tolerance to metal ions, and many common metal ions, such as K^+^, Ca^2+^, Mg^2+^, Co^2+^, and Fe^3+^, can promote the activity of AlyRm3. To the best of our knowledge, AlyRm3 is the most active thermophilic type of alginate lyase found to date and maintains its activity in the presence of a variety of metal ions, making it advantageous as an industrial enzyme. A medium-scale experiment of saccharification of sodium alginate using AlyRm3 yielded alginate oligosaccharides at a concentration of 1.73 g/L in just two hours of reaction time. The results of the saccharification experiments further demonstrated the industrial value of AlyRm3 for the production of alginate oligosaccharides.

In addition, AlyRm3 uses the endo-mode to degrade alginate. AlyRm3 preferentially degrades alginate but also shows high polyM (28,814.31 U/mg) and polyG (21,329.21 U/mg) degradation activity compared to other alginate lyases. The enzyme kinetic constants (*K_cat_, K_m_*, and *V_max_*) of AlyRm3 demonstrated that this lyase has the strongest affinity for the G-blocks and the highest catalytic efficiency for the M-blocks. Early in the reaction, the three substrates can be degraded into tetrasaccharides, trisaccharides, and disaccharides, and as the progress of the reaction, the tetrasaccharides can be degraded into trisaccharides and disaccharides. Based on the homology model and sequence alignment, we can observe that the substrate-binding cleft is open at both ends, such that longer chain polysaccharides can be accommodated by the enzyme, which we speculate may be one of the factors contributing to its high enzymatic activity. The excellent thermophilicity and temperature stability of AlyRm3 are speculated to be due to the structural rigidity supported by the α-helix in its structure. A more in-depth study of AlyRm3, especially the structural basis, will provide a solid foundation for the analysis and understanding of the thermo-stable mechanism. With the increasing demand for alginate oligosaccharides in the market, alginate lyase with high stability and high enzymatic activity will be the preferred tool for the industrial production of alginate oligosaccharides. In summary, the excellent enzymatic properties of AlyRm3 make it a potentially promising biological tool that is well-suited for tailoring alginate oligosaccharides.

## 4. Experimental Materials and Methods

### 4.1. Sequence Analysis

The structural domain of AlyRm3 was analyzed using CD-Search Results in NCBI (https://www.ncbi.nlm.nih.gov/Structure/cdd/wrpsb.cgi accessed date: 23 November 2022). Multiple sequence alignment of the full sequence of AlyRm3 with the already characterized alginate lyases was performed using Vector NTI. A phylogenetic tree of related protein sequences from PL 6, 17, and 39 families was constructed using the MEGA 6.0 software. According to the structure of alginate lyase Dp0100 from *Defluviitalea phaphyphila* (PDB: 6JPN), the three-dimensional structure of AlyRm3 was constructed through SWISS-MODEL (https://swissmodel.expasy.org/ accessed date: 23 November 2022). All structures appearing in this paper were visualized by the PyMOL software (PyMOL2.5, Schrödinger).

### 4.2. Cloning, Heterologous Expression, and Purification of AlyRm3

The gene AlyRm3 (GenBank accession. CP001807) was synthesized by GENEWIZ (Suzhou, China), based on the predicted sequence of the alginate lyase gene from *Rhodothermus marinus* DSM 4252. This gene was ligated into the pET-21a (+) vector. The synthetic plasmid was obtained and transformed into receptor cells *E. coli* BL21 (DE3). The recombinant strain was grown in Luria-Bertani (LB) including 100 μg/mL ampicillin and was incubated at 37 °C for 10–12 h, and it induced expression of the AlyRm3 at 22 °C for 36–48 h after the addition of 0.1 mM IPTG. After protein expression was completed, the AlyRm3 expressed by the bacterium was collected and the crude enzyme solution obtained was purified by Ni-NTA sepharose column (GE Healthcare, Uppsala, Sweden) and Amicon Ultra-15mL, 50 kDa Centrifugal Filter Unit (Millipore, Shanghai, China) [24]. SDS-PAGE was applied to analyze the purity of the recombinant AlyRm3. Furthermore, the Coomassie brilliant blue G-250 (Beyotime Institute of Biotechnology, Nantong, China) was used to test the concentration of proteins.

### 4.3. Substrate Specificity and Enzyme Kinetic Constants

The activity of AlyRm3 was investigated using the ultraviolet absorption method described by Cao [25]. The 10-fold dilution of 50 μL purified proteins was incubated with 150 μL 0.5% (*w*/*v*) of three substrates dissolved in 10 mM Tris-HCl buffer pH 8.0 at 27 °C for 15 min. These three substrates are the sodium alginate from *Macrocystis pyrifera* which was purchased from Sigma-Aldrich (M/G ratio 77/23, viscosity ≥ 2000 Cp, St. Louis, MO, USA), PolyM and polyG (purity: about 95%, M/G ratio: 97/3 and 3/97, respectively), which were purchased from Qingdao BZ Oligo Biotech Co., Ltd. (Qingdao, China). This paper defined one-unit enzymatic activity as the amount of enzyme required to increase the absorbance at 235 nm (extinction coefficient: 6150 M^−1^·cm^−1^) by 0.1 per min. The kinetic constants of AlyRm3 towards three substrates were investigated by measuring the degradation activity of AlyRM3 on different concentrations (0.2–10 mg/mL) of substrates. The enzyme kinetic constants (*K_cat_, K_m_*_,_ and *V_max_* values) of AlyRm3 were calculated as reported by Zhu et al. [26].

### 4.4. Biochemical Characterization of AlyRm3

To determine the optimal temperature and the thermal stability of AlyRm3, reactions were carried out at 35–90 °C. Additionally, the recombinant AlyRm3 was incubated at 65–80 °C for 0–60 min and the remaining enzymatic activity was tested to investigate the thermostability of the enzyme. The effect of pH on the enzymatic activity of AlyRm3 was investigated by testing the activity of AlyRm3 present in different pH buffers (50 mM Na_2_HPO_4_-citric acid buffer (pH 3.0–5.0), 50 mM NaH_2_PO_4_-Na_2_HPO_4_ (pH 6.0–8.0), 50 mM Tris-HCl (pH 8.0–9.0), 50 mM glycine-NaOH (pH 9.0–10.0), and 50 mM Na_2_HPO_4_-NaOH (pH 11.0–12.0)). Moreover, the purified AlyRm3 was incubated with different pH buffers at 4 °C for 24 h, and the pH stability of the AlyRm3 was characterized by measuring the residual activity of the AlyRm3 after incubation. Furthermore, to investigate the effects of metal ions on the enzymatic activities of AlyRm3, the purified AlyRm3 was mixed with 0.5% sodium alginate at a ratio of 1:3, and various metal ions at a final concentration of 1mM were added to the reaction system. The reaction was performed at 70 °C for 30 min, and the residual activity of AlyRm3 was measured. The reaction system without adding any metal ions was used as the control group (100%). To ensure the accuracy of the experiments, all experiments were performed with three replicates.

### 4.5. Products Distribution and Action Pattern Analysis of AlyRm3

To determine the smallest substrate of AlyRm3, AlyRm3, and OligoM (10 mg/mL) with different DPs (DP2–7) purchased from Qingdao BZ Oligo Biotech Co., Ltd. (Qingdao, China) were mixed in 10 mM Tris-HCl buffer (pH 8.0) and reacted at 70 °C for 24 h. In addition, to explore the action pattern and degradation products of AlyRm3, 100 μL purified AlyRm3 (0.9058 mg/mL) was incubated with 300 μL 0.5% (*w*/*v*) three substrates (sodium alginate, polyM, and polyG) at 70 °C for 0–72 h and sampled after 0, 5, 15, 30 min, 1, 2, 6, 12, 24, 48 h, and 72 h, respectively. The degradation products of AlyRm3 were analyzed by using fast protein liquid chromatography equipped with Superdex peptide 10/300 GE Column (GE Health), as reported by Li et al. [27]. In addition, ESI−MS analyzed the end products of AlyRm3 in a positive-ion mode using the following settings, as previously reported [27].

### 4.6. Saccharification of Alginate by AlyRm3

To determine the saccharification potential of AlyRm3 on alginate, the purified enzyme (0.2981 mg/mL) was added to the buffer (10 mM Tris-HCl, 300 mM NaCl, pH 8.0) at a ratio of 1% (*v*/*v*), followed by sodium alginate solid particles at a ratio of 0.5% (*w*/*v*). The saccharification reaction was carried out at 200 rpm and 70 °C. Samples were taken respectively after reaction for 30, 40, 50, 60, 80, and 120 min, and the viscosity of the samples was measured with SNB-3 digital viscometer (Shjingmi, Shanghai, China). Samples were taken after reaction for 1, 2, 3, 4, 6, 8, 10, and 12 h, respectively, and the reducing sugar content of the samples was measured through 3,5-dinitrosalicylic acid (DNS) colorimetry using glucose as the standard [28].

## Figures and Tables

**Table 1:** Substrate specificity and kinetics of AlyRm3.

Substrate	Sodium Alginate	PolyM	PolyG
Activity (U/mg)	37,315.08	28,814.31	21,329.21
*K_m_*(mM)	15.6738	7.3264	5.3961
*V_max_* (mol/s)	0.3285	0.5446	0.0497
*k_cat_* (s^−1^)	141.69	2323.78	212.07
*k_cat_*/*K_m_* (s^−1^/mM)	89.4288	317.1783	39.3006

## Data Availability

Not applicable.

## Supplementary Materials

The following supporting information can be downloaded at: https://www.mdpi.com/article/10.3390/md21030180/s1. The sequence analysis of AlyRm3 is shown in Figures S1 and S2. The summary of some alginate lyases with high activity is shown in Table S1. The summary of some alginate lyases with thermophilic features is shown in Table S2. References [29,30,31,32,33,34,35,36,37,38,39,40] are cited in the supplementary materials.
