# Supplementary material for: Efficient Degradation of Alginate and Preparation of Alginate Oligosaccharides by a Novel Biofunctional Alginate Lyase with High Activity and Excellent Thermophilic Features
Source: Mar Drugs. 2023 Mar 14;21(3):180. doi: 10.3390/md21030180 (PMC10056287; doi:10.3390/md21030180)
Supplement: Supplementary file 1 [file marinedrugs-21-00180-s001.zip › marinedrugs-2276636-supplementary.pdf]

Supplementary Materials

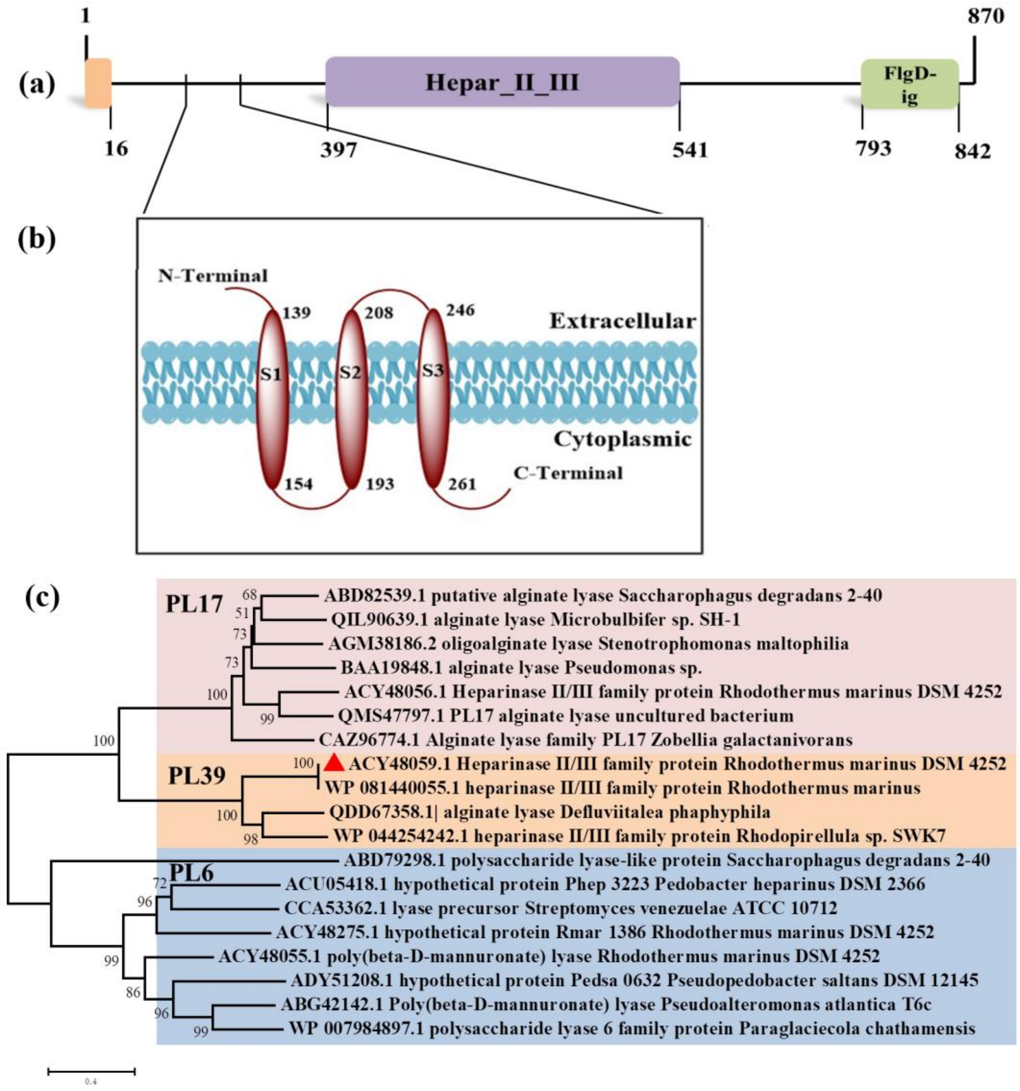

Figure S1. The domain analysis and phylogenetic analysis of AlyRm3. (**a**) The domain of AlyRm3. (**b**) The transmembrane helices in AlyRm3. (**c**) The phylogenetic analysis of AlyRm3 with other alginate lyases. AlyRm3 is indicated by a red triangle. The tree is drawn to scale, with branch lengths in the same units as those of the evolutionary distances used to infer the phylogenetic tree.

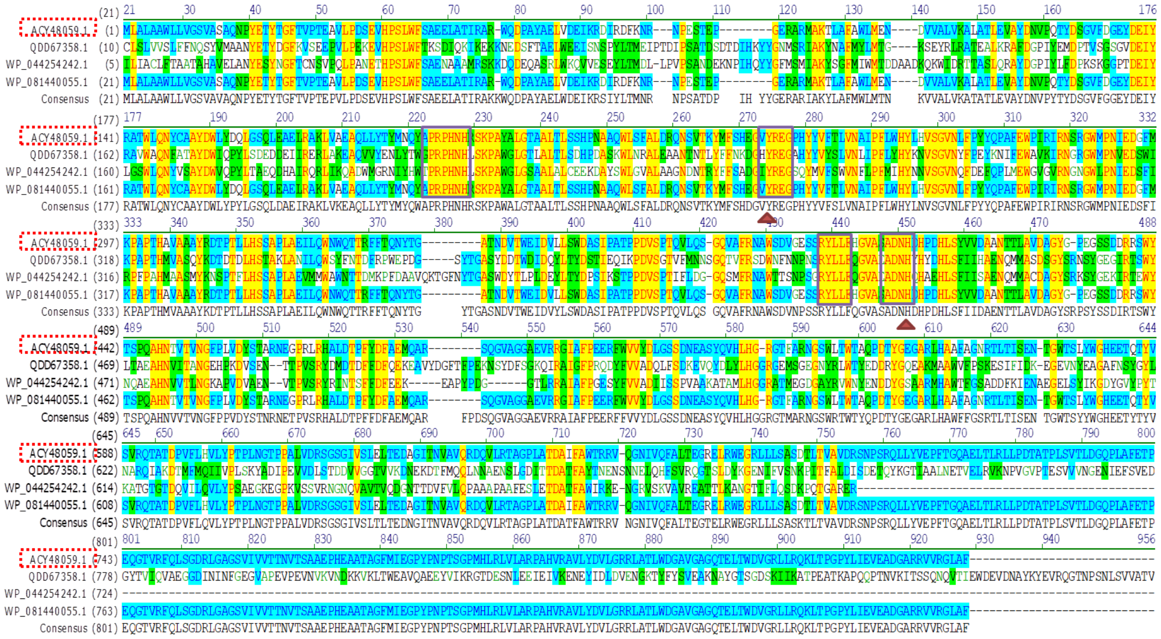

Figure S2. The multiple amino-acid sequence alignment of AlyRm3 and other alginate lyases of the PL39 family. QDD67358.1 from *Defluviitalea phaphyphila*. WP_044254242.1 from *Rhodopirellula* sp. SWK7. WP_081440055.1 from *Rhodothermus marinus*. Shaded yellow indicate identical and similar amino-acid residues in alginate lyase. The violet boxes indicate the positions of the three conserved regions. Red triangles are catalytic acid and catalytic base. The figure was prepared using Vector NTI.

Table S1. The summary of some alginate lyases with high activity.

Enzyme	Organisms	Family(PL)	Substrate specificities	activity	Reference
AlyRm3	*Rhodothermus marinus* DSM 4252	39	bifunction	sodium alginate:37315.08 U/mg#	This study
PsMan8A	*Paradendryphiella salina*	8-4	only acts on polyM	polyM:1093 ± 17 U/mg#	[28]
Aly7A	*Vibrio* sp. W13	7	bifunction	alginate:15718 U/mg#	[29]
Dp0100-TM5	*Defluviitalea phaphyphila*	39	polyG	polyG: 16058±136#	[16]
Alys1	*Tamlana* sp. s12	7	polyM	alginate:1350 U/mg*	[30]
AlgNJU-03	*Vibrio* sp. NJU-03	7	bifunction	alginate:6468.99 U/mg#	[31]
AlgA	*Bacillus* sp. Alg07	7	polyM	alginate: 8306.7 U/mg#	[32]
AlgNJ-07	*Serratia marcescens* NJ-07	NA	polyM	alginate: 2742.5 U/mg#	[33]
AlySY08	*Vibrio* sp. SY08	NA	polyG	alginate: 1183.7 U/mg#	[34]

It should be noted that the activity of each enzyme is defined differently. #: One unit enzymatic activity was defined as the amount of enzyme required to increase the absorbance at 235 nm by 0.1 per min.*: One unit (U) of enzyme activity was defined as the amount of enzyme required to release 1 μmol of reducing sugar per min.

Table S2. The summary of some alginate lyases with thermophilic features.

**Enzyme**	**Organisms**	**Family(PL)**	**Optimum temperature/pH**	**Temperature stability**	**Reference**
AlyRm3	*Rhodothermus marinus* DSM 4252	39	70℃/8.0	Retain almost all of the maximum activity after 1 hour incubation at 60℃	This study
Alg823	*Pseudomonas carrageenovora* ASY5	6	55℃/8.0	Retain over 75.0% of the maximum activity after 30 min of incubation at 50°C	[35]
rNitAly	*Nitratiruptor* sp. SB155-2	7	70℃/6.0	Retain over 50% of the maximum activity after 30 min of incubation at 67℃	[17]
Dp0100-TM5	*Defluviitalea phaphyphila*	39	65℃/5.8	/	[16]
Alg7D	*Saccharophagus degradans* 2-40	7	50℃/7.0	The activity decreased by 16% after 15 min pre-incubation at 50℃	[36]
AlgL	*Sphingomonas* sp. MJ-3	17	50℃/6.5	/	[37]
PsAly	*Paenibacillus* sp. str. FPU-7	31	65℃/7-7.5	Retain 80% of the maximum activity after 60 min of incubation at 37℃	[38]
AMOR_PL17A	Ridge (AMOR) metagenomics data set	17	90℃/5.0	Retained 100% activity after a 24 h incubation at 60℃	[15]
AlyM	*Microbulbifer* sp. Q7	7	55℃/7.0	AlyM was stable at a temperature of no more than 40℃	[39]
FlAlyA	*Flavobacterium* sp. UMI-01	7	55℃/7.7	Retain 20% of activity after 30 min of incubation at 50℃	[40]
